# Supplementary material for: Excitation and inhibition in anterior cingulate predict use of past experiences
Source: eLife. 2017 Jan 5;6:e20365. doi: 10.7554/eLife.20365 (PMC5213710; doi:10.7554/eLife.20365)
Supplement: Figure 3—source data 2. — The maps are in NIfTI format and can be opened with freely available data viewers such as FSLView or MRIcron. DOI: http://dx.doi.org/10.7554/eLife.20365.012 [file elife-20365-fig3-data2.zip › Figure3_SourceData2_updated/Figure3_SourceData2_Legend.rtf]

bg_image.nii.gz: average structural imageLearntInformation_Main_activation: activations with information to be learnt at the time of the outcome, i.e. chosen reward magnitude outcome - chosen effort magnitude outcome - unchosen reward magnitude outcome + unchosen effort magnitude outcome; thresholded using whole-brain cluster-correction (p<0.05, voxel-inclusion threshold: z>2.3). See also GLM1 in the main text methods.LearntInformation_Main_deactivation: deactivations with information to be learnt at the time of the outcome; thresholded using whole-brain cluster-correction (p<0.05, voxel-inclusion threshold: z>2.3)LearntInformation_Main_nonthresh: activations and deactivation, without correction for multiple comparison (non-thresholded)LearntInformation_BehavCovariate: contrast map of the relationship between the behavioural covariate (one value per participant), i.e. the use of learnt information from the computational model, and the brain signal to the information to be learnt (whole-brain cluster-corrected p<0.05, voxel-inclusion threshold: z>2.3). Clusters show areas in which stronger neural representation of information to be learnt across people relates to stronger behavioural use of the learnt information. See also GLM2 in the main text methods.LearntInformation_BehavCovairate_nonthresh: same as above, without correction for multiple comparisonLearntInformation_SpectrCovariate: contrast map of relationship between Glutamate-GABA levels and brain signal to the learnt information (cluster-corrected in anatomical region of interest from spectroscopy mask p<0.05, voxel-inclusion threshold: z>2.3). Cluster shows areas in which stronger neural representation of information to be learnt across people relates to higher Glu-GABA. See also GLM3 in the main text methods.LearntInformation_SpectrCovariate_nonthesh: same as above, without correction for multiple comparisons
